# Coagulation and heparin requirements during ablation in patients under oral anticoagulant drugs

**DOI:** 10.1002/joa3.12357

**Published:** 2020-05-19

**Authors:** Philippe Maury, Slimane Belaid, Agnès Ribes, Quentin Voglimacci‐Stephanopoli, Pierre Mondoly, Marie Blaye, Franck Mandel, Benjamin Monteil, Didier Carrié, Michel Galinier, Vanina Bongard, Anne Rollin, Sophie Voisin

**Affiliations:** ^1^ Department of Cardiology University Hospital Rangueil Toulouse France; ^2^ Unité INSERM U 1048 Toulouse France; ^3^ Hematology laboratory University Hospital Rangueil Toulouse France; ^4^ USMR (Unité de Soutien méthodologique à la recherche) University Hospital Rangueil Toulouse France; ^5^ UMR 1027 INSERM‐Université Toulouse 3 France

**Keywords:** ablation, activated clotting time, anticoagulation, direct oral anticoagulant

## Abstract

**Background:**

Anticoagulation during catheter ablation should be closely monitored with activated clotting time (ACT). However vitamin K antagonists (VKA) or direct oral anticoagulant drugs (DOAC) may act differently on ACT and on heparin needs. The aim of this study was to compare ACT and heparin requirements during catheter ablation under various oral anticoagulant drugs and in controls.

**Methods:**

Sixty consecutive patients referred for ablation were retrospectively included: group I (n = 15, VKA), group 2 (n = 15, uninterrupted rivaroxaban), group 3 (n = 15, uninterrupted apixaban), and group 4 (n = 15, controls). Heparin requirements and ACT were compared throughout the procedure.

**Results:**

Heparin requirements during the procedure were significantly lower in patients under VKA compared to DOAC, but similar between DOAC patients and controls.

Activated clotting time values were significantly higher in patients under VKA compared to DOAC and similar in DOAC patients versus controls. Furthermore, anticoagulation control as evaluated by the number/proportion of ACT> 300 as well as the time passed over 300 seconds was significantly better in patients under VKA versus DOAC, without significant differences between DOAC and controls. Finally, the number of patients/ACT with excessive ACT values was significantly higher in VKA versus DOAC patients versus controls.

There was no significant difference between rivaroxaban and apixaban for ACT or heparin dosing throughout the procedure.

**Conclusion:**

Vitamin K antagonists allowed less heparin requirement despite reaching higher ACT values and more efficient anticoagulation control (with more excessive values) compared to patients under DOAC therapy and to controls. There was no difference in heparin requirements or ACT between DOAC patients and controls.

## Graphical abstract

VKA allowed less heparin requirement despite reaching higher ACT values and more efficient anticoagulation control (although with a higher risk for excessive values) compared to patients under long‐term direct anticoagulant therapy and to controls referred for ablation Activated clotting time may not be a sensitive marker of anticoagulation in patients under direct anticoagulant drugs and heparin

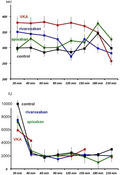

## 1 INTRODUCTION

Percutaneous catheter ablation has become the therapeutic of choice for patients with cardiac arrhythmias and the number of worldwide ablation procedures has dramatically increased over the previous years.^1^

Anticoagulation is usually required during percutaneous catheter ablation procedures, because of the risk of material and lesion‐induced cardiac thrombus and embolism.^2^, ^3^ This thrombo‐embolic risk persists even in patients under oral anticoagulation, thus additional anticoagulation with unfractionated heparin is needed.^4^

Because of combined risks of bleeding and thrombo‐embolic events, it is furthermore recommended that anticoagulation levels should be closely monitored during ablation procedures: achieving and maintaining activated clotting time (ACT) > 300 seconds with heparin is currently considered mandatory for ablation of atrial fibrillation^5^ and also commmonly used for ablation of ventricular arrhythmias.^6^ Activated clotting time is a widely accepted coagulation monitoring technique^3^, ^4^, ^7^ being perfectly adapted to clinical requirements, since results are very quickly available using bedside coagulation monitoring, allowing immediate adjustment of heparin needs.^8^

In patients under long‐term oral anticoagulation, ablations are optimally conducted without any interruption of anticoagulant drugs.^3^ Uninterrupted use of warfarin has been previously recommended.^6^ Feasibility and safety of uninterrupted direct acting oral anticoagulant (DOAC) on periprocedural periods of ablation has more recently been demonstrated^9^, ^10^, ^11^ and is currently also recommended in the most recent guidelines.^6^

Vitamin K antagonists (VKA) or DOAC may act differently on ACT, and thus on heparin needs for achieving ACT target values. ACT and response to heparin have been shown to be different between patients receiving VKA or DOAC.^12^, ^13^, ^14^ Furthermore, target ACT value in patients on uninterrupted DOAC is based on very limited evidence, mostly extrapolated from VKA‐treated populations. Nonetheless, differences in ACT and in heparin requirements according to the type of oral anticoagulant drug remain poorly investigated. Moreover no comparison with controls (ie, without previous oral anticoagulation) is currently available.

The aim of this study was to investigate and compare the ACT values and the requirement of heparin in patients undergoing percutaneous catheter ablation under various oral anticoagulant drug regimens and in controls.

## 2 METHODS

Sixty consecutive patients referred for ablation of left atrial or ventricular arrhythmias over the last six months at our center were retrospectively included. Patients were divided in four groups: group I (n = 15, patients under long‐term VKA therapy), group 2 (n = 15, long‐term rivaroxaban), group 3 (n = 15, long‐term apixaban), and group 4 (n = 15, controls, no previous oral or intravenous/subcutaneous anticoagulation). The number of included patients in each group was similar for statistical purposes, and inclusions were consecutive, not random. Every patient from group 1 to 3 were on long‐term oral anticoagulation therapy (for >2 months), with INR in the therapeutic range (group 1) or without any missing drug intake during the weeks before ablation (groups 2 and 3).

Patients underwent radio‐frequency or cryo‐ablation for left atrial arrhythmias (n = 45, groups 1 to 3) or ventricular arrhythmias (n = 15, controls). In every patient undergoing left atrial ablation, transoesophageal echocardiography ruled out atrial thrombus before the procedure. Values of each ACT and heparin dosing could be retrieved from our electrophysiological laboratory files since all these data were kept together with precise timings.

Automated ACT measurements were done using Hemochron **®** Jr Signature + device (Accriva Diagnostics, San Diego, CA). No baseline ACT had been performed, but baseline prothrombin time and activated partial thromboplastin time (aPTT) were available in all patients. Hemostasis assays were performed using Neoplastine CI+ (prothrombin time) and CK Prest on Star Evolution (aPTT) both from STAGO (France).

Intravenous unfractionated heparin was infused as soon as the transseptal access was achieved (for patients with left atrial ablation or when transseptal access was needed for left ventricular ablation) or after arterial vascular access (in case of retrograde aortic access for left ventricular ablation). Initial heparin dosing was determined according to the current oral anticoagulation and/or weight (usually 5000‐7000 IU for patients under VKA and between 5000 and 10 000 IU for DOAC patients, based on weight and on our previous experience, or 100 IU/kg for non anticoagulated patients). First ACT was checked 20 minutes after the initial heparin infusion, then rechecked again 20 minutes later, once again 20 minutes later, then every 30 minutes. Target ACT value was 300 seconds in all patients.^3^, ^4^ Any ACT < 300 seconds was leading to new heparin infusion (dosing according to the ACT value, from 1000 to 3000 U).

The total amount of infused heparin during the whole procedure was calculated, as well as the time and heparin dosing needed before achieving ACT value of 300 seconds, the time passed over 300 seconds, the number/proportion of ACT ≥ 300 seconds, number/proportion of ACT and patients with ACT ≥ 400 seconds, number/proportion of ACT or patients with ACT < 300 after being ≥ 300 seconds.

Protamine was infused at the end of the procedure prior to removal of vascular sheaths in each patient (dosing according to the total heparin delivered). Hemorragic or embolic complications were noted.

Informed consent was obtained from all patients. This study was approved by Toulouse University Hospital completing standard ethic requirements, and declared to the CNIL (Commission Nationale de l'Informatique et des Libertés) according to the French law and cover by the MR‐004 (CNIL number: 2206723 v 0) on 13 september 2018.

## 3 STATISTICS

Continuous variables are reported as median and IQR and compared with nonparametric tests (Mann Whitney when comparing two groups and Kruskal‐Wallis when comparisons included more than two groups). Categorical variables were compared using Fischer's exact test. Comparisons of repeated ACT and of repeated heparin dosings between goups were performed using repeated measure ANOVA when there was sufficient number of values. Multivariate analysis was performed by logistic regression using variables significantly associated in univariate analysis, leading to calculation of odds ratio (OR) and 95% confidence intervals (CI).

Analysis and calculations were performed using StatView ™ program (Abacus Concepts, Inc Berkeley, CA 1992‐1996, version 5.0). A *P* < .05 was considered statistically significant for each analysis.

## 4 RESULTS

### 4.1 Clinical characteristics

Clinical characterictics of the patient population are depicted in Table 1. Drugs used in group 1 were warfarin (n = 3), fluindione (n = 11) and acecoumarol (n = 1). All patients had INR in the therapeutic range before ablation. There was no missing drug intake during the weeks before ablation in groups 2 and 3 patients, and DOACs were “truly” uninterrupted before the procedure (not any discontinuation in any patient even the day of the procedure). Drug dosing in groups 2 and 3 were considered optimal according to age, weight and renal function: 20 mg rivaroxaban daily in each and 5 mg bid apixaban in each but one 82‐year‐old patient weighing 56 kg (2.5 mg bid). Data from this patient were not different from other group 3 patients, thus they were retained for analysis.

**TABLE 1:** Clinical characterictics of the patient population

Males	40/60 (66%)
Age (y)	60 (IQR 17)
Weight (kg)	79 (IQR 24)
Height (cm)	174 (IQR 12)
Body mass index (BMI) (kg/m^2^)	25 (IQR 5)
Body surface (m^2^)	1.9 (IQR 0.3)
LVEF (%)	50 (IQR 15)
Creatinine (μm/L)	87 (IQR 33)
Glomerular filtration (mL/min/kg)	76 (IQR 31)
Left atrial surface (cm^2^)	24 (IQR 23)
Left atrial volume (mL/m^2^)	55 (IQR 35)
CHA2Ds2VaSC^a^	2 (IQR 2.2)
Hypertension	19/45 (42%)
Diabetes	9/45 (20%)
Heart failure	10/45 (22%)
Previous stroke/embolism	7/45 (16%)
Vascular disease	13/45 (29%)

^a^ CHA2Ds2VaSC score and items not evaluated in the control group.

Comparisons between group 2 and 3 failed to demonstrate any significant difference, except for a trend towards higher CHA2DS2VaSC score in the rivaroxaban group (median 3, IQR 2.7) compared to that in apixaban group (median 1, IQR 1.7) (*P* = .09) (Table 2). Thus, group 2 and 3 were mixed (DOAC group) and compared to group 1 (VKA) and controls (Table 3).

**TABLE 2:** Comparisons rivaroxaban and apixaban

	Rivaroxaban	Apixaban	*P* value
Male gender	14/15 (93%)	12/15 (80%)	*P* = ns
Age (y)	65 (IQR 16)	58 (IQR 21)	*P* = ns
Weight (kg)	78 (IQR 14)	76 (IQR 32)	*P* = ns
Height (cm)	174 (IQR 9)	173 (IQR 20)	*P* = ns
BMI (kg/m^2^)	28 (IQR 4.7)	24 (IQR 5.1)	*P* = ns
Body surface (m^2^)	1.9 (IQR 0 0.13)	1.9 (IQR 0.47)	*P* = ns
LVEF (%)	55 (IQR 9)	50 (IQR 16)	*P* = ns
CHA2DS2VaSC	3 (IQR 2.75)	1 (IQR 1.75)	.09
Creatinine **(μ**m/L)	88 (IQR 23)	83 (IQR 33)	*P* = ns
Glomerular filtration (mL/min/kg)	77 (IQR 24)	77 (IQR 30)	*P* = ns
Left atrial surface (cm^2^)	26 (IQR 12)	34 (IQR 13)	*P* =ns
Left atrial volume (mL/m^2^)	51 (IQR 22)	77 (IQR 24)	*P* = ns

**TABLE 3:** Comparisons VKA‐DOAC‐controls

	VKA	DOAC	Controls	3 groups	VKA versus DOAC	DOAC versus controls	VKA versus controls
Male gender	9/15 (60%)	26/30 (87%)	15/15 (100%)	*P* = .01	*P* = .05	*P* = ns	*P* = .006
Age (y)	69 (IQR 16)	61 (IQR 17)	56 (IQR 14)	*P* = ns	*P* = ns	*P* = ns	*P* = .05
Weight (kg)	76 (IQR 26)	77 (IQR 19)	89 (IQR 32)	*P* = .05	*P* = ns	*P* = .02	*P* = .06
Height (cm)	171 (IQR 13)	173 (IQR 10)	178 (IQR 12)	*P* = ns	*P* = ns	*P* = ns	*P* = ns
BMI (kg/m^2^)	25 (IQR 5)	25 (IQR 6)	26 (IQR 5)	*P* = ns	*P* = ns	*P* = ns	*P* = ns
Body surface (m^2^)	1.9 (IQR 0.3)	1.9 (IQR 0.3)	2 (IQR 0.3)	*P* = ns	*P* = ns	*P* = ns	*P* = ns
LVEF (%)	50 (IQR 10)	55 (IQR 15)	50 (IQR 24)	*P* = ns	*P* = ns	*P* = ns	*P* = ns
CHA2DS2VaSC	3 (IQR 2)	2 (IQR 2)	—	—	*P* = .04	—	—
Creatinine (μm/L)	93 (IQR 77)	85 (IQR 29)	82 (IQR 31)	*P* = ns	*P* = ns	*P* = ns	*P* = ns
Glomerular filtration (mL/min/kg)	59 (IQR 44)	77 (IQR 29)	88 (IQR 31)	*P* = .03	*P* = .04	*P* = ns	*P* = .02
Left atrial surface (cm^2^)	33 (IQR 26)	21 (IQR 18)	—	—	*P* = ns	—	—
Left atrial volume (mL/m^2^)	44 (IQR 48)	66 (IQR 30)	—	—	*P* = ns	—	—

Controls were heavier compared to VKA and DOAC. There were more women in the VKA group compared to DOAC and controls. CHA2DS2VaSC score was higher in the VKA group as a result of more stroke and women in this group and bordeline older age. Finally, glomerular filtration was lower in VKA patients compared to DOAC and controls.

### 4.2 Coagulation and heparin requirements

Except for longer baseline aPTT in patients with rivaroxaban (median 1.2, IQR 0.1) compared to apixaban patients (median 1.1, IQR 0.1) (*P* = .005), there was no difference between group 2 and 3 patients for any coagulation time or heparin dosing at baseline or throughout the procedure (Table 4). Thus, group 2 and 3 were mixed (DOAC group) and compared to group 1 (VKA) and controls. Coagulation parameters and heparin requirements in patients under VKA, DOAC and controls are depicted in Table 5.

**TABLE 4:** Coagulation parameters and heparin requirements in rivaroxaban and apixaban patients

	Rivaroxaban	Apixaban	*P* value
Prothrombin time (s)	18 (IQR 4.5)	15 (IQR 4)	*P* = ns
aPTT (ratio vs. control)	1.22 (IQR 0.1)	1.1 (IQR 0.12)	*P* = .005
Heparin T0 (IU)	70 (IQR 10)	70 (IQR 20)	*P* = ns
Total heparin (IU)	100 (IQR 57)	110 (IQR 57)	*P* = ns
Heparin before ACT > 300 (IU)	80 (IQR 30)	80 (IQR 40)	*P* = ns
Delay before ACT > 300 (min)	20 (IQR 31)	35 (IQR 35)	*P* = ns
Duration with ACT > 300 (min)	70 (IQR 81)	40 (IQR 75)	*P* = ns
nb ACT > 300	3 (IQR 3.5)	2 (IQR 1.7)	*P* = ns
Proportion ACT > 300 (%)	57 (IQR 35)	50 (IQR 33)	*P* = ns
Patients with ACT < 300 after ACT > 300	10/15 (66%)	13/15 (87%)	*P* = ns
nb ACT < 300 after > 300	1 (IQR 2.7)	1 (IQR 1.7)	*P* = ns
Proportion ACT < 300 after > 300 (%)	15 (IQR 55)	33 (IQR 13)	*P* = ns
Patients with ACT > 400	8/15 (53%)	10/15 (66%)	*P* = ns
nb ACT > 400	0 (IQR 1)	0 (IQR 1)	*P* = ns
Proportion ACT > 400 (%)	0 (IQR 27)	0 (IQR 16)	*P* = ns
Protamine (AHU)	100 (IQR 26)	100 (IQR 45)	*P* = ns
Procedural duration (min)	210 (IQR 82)	240 (IQR 101)	*P* = ns

**TABLE 5:** Coagulation parameters and heparin requirements in the three groups

	VKA	DOAC	Controls	3 groups	VKA versus DOAC	DOAC versus controls
Prothrombin time (s)	27 (IQR 6)	16 (IQR 4)	14 (IQR 2)	*P* < .0001	*P* < .0001	*P* = .001
aPTT (ratio vs. `control)	1.2 (IQR 0.2)	1.1 (IQR 0.6)	1 (IQR 0.15)	*P* < .0001	*P* = .02	*P* = .0005
Heparin T0 (IU)	5000 (IQR 2000)	7000 (IQR 2000)	10 000 (IQR 3700)	*P* < .0001	*P* = .007	*P* = .0002
Heparin T0 (IU/kg)	0.74 (IQR 0.13)	1 (IQR 0 0.36)	1.12 (IRQ 0.27)	*P* = .001	*P* = .003	*P* = .08
Total heparin (IU)	7000 (IQR 1000)	11 000 (IQR 6000)	14 000 (IQR 7200)	*P* < .0001	*P* = .0009	*P* = .03
Total heparin (IU/kg)	0.87 (IQR 0.37)	1.52 (IQR 0.55)	1.5 (IQR 0.83)	*P* = .001	*P* = .0006	*P* = ns
Total heparin (IU/h)	20 (IQR 10)	30 (IQR 11)	31 (IQR 11)	*P* = .02	*P* = .006	*P* = ns
Heparin before ACT > 300 (IU)	7000 (IQR 2000)	8000 (IQR 3000)	12 000 (IQR 2000)	*P* < .0001	*P* = .002	*P* = .003
Heparin before ACT > 300 (IU/kg)	0.76 (IQR 0.2)	1.16 (IQR 0.28)	1.23 (IQR 0.43)	*P* = .0004	*P* = .0005	*P* = ns
Delay before ACT > 300 (min)	20 (IQR 3.7)	20 (IQR 35)	20 (IQR 30)	*P* = ns	*P* = ns	*P* = ns
Duration with ACT > 300 (min)	150 (IQR 82)	50 (IQR 90)	30 (IQR 67)	*P* = .0004	*P* = .0005	*P* = ns
nb ACT > 300	5 (IQR 2.7)	2.5 (IQR 2)	1 (IQR 3)	*P* = .003	*P* = .009	*P* = .1
Proportion ACT > 300 (%)	83 (IQR 25)	53 (IQR 38)	37 (IQR 49)	*P* = .0002	*P* = .0009	*P* = .08
Patients with ACT < 300 after ACT > 300	9/15 (60%)	23/30 (77%)	11/15 (73%)	*P* = ns	*P* = ns	*P* = ns
nb ACT < 300 after > 300	1 (IQR 1)	1 (IQR 2)	1 (IQR 1.7)	*P* = ns	*P* = .08	*P* = ns
Proportion ACT < 300 after > 300 (%)	14 (IQR 19)	25 (IQR 39)	20 (IQR 47)	*P* = ns	*P* = .04	*P* = ns
Patients with ACT > 400	12/15 (80%)	12/30 (40%)	1/15 (7%)	*P* = .0002	*P* = .01	*P* = .02
nb ACT > 400	2 (IQR 2.7)	0 (IQR 1)	0 (IQR 0)	*P* = .0004	*P* = .003	*P* = .06
Proportion ACT > 400 (%)	33 (IQR 57)	0 (IQR 20)	0 (IQR 0)	*P* = .0005	*P* = .003	*P* = .07
Protamine (AHU)	100 (IQR 40)	100 (IQR 30)	100 (IQR 0)	*P* = ns	*P* = ns	*P* = ns
Procedural duration (min)	210 (IQR 112)	225 (IQR 80)	280 (IQR 52)	*P* = .02	p = ns	*P* = .0009

Baseline prothrombin time and aPTT were significantly lower in controls compared to DOAC, which were also significantly lower compared to VKA patients. Duration of the procedure was longer in controls but did not differ between VKA and DOAC patients.

Initial heparin dosing, dosing till achievement of ACT > 300 and total heparin requirements were significantly higher in controls versus DOAC patients, which were also significantly higher compared to VKA patients. However, dosings of heparin expressed in IU/kg or in IU/h were no more significantly different between DOAC patients and controls.

First ACT was significantly longer in VKA (median 400 msec, IQR 123) versus DOAC patients (305, IQR 83) (*P* = .02) but similar in DOAC versus controls (305, IQR 93) (p = ns). The percentage of patients in whom ACT after the first heparin infusion was >300 seconds was 87% in VKA patients versus 60% in DOAC patients (*P* = .06) and 53% in controls (ns between DOAC and controls), while delays before reaching the target value of 300 seconds did not differ between groups. Averaged ACT values during the whole procedure were longer in VKA (330 msec, IQR 35) versus DOAC patients (297 ms, IQR 33) (*P* = .003), which were similar to that of controls (298 ms, IQR 69) (*P* = ns).

ACT > 300 was reached at least one time during the procedure in every patient but one in the apixaban group and three controls (*P* = .05). Number and proportion of ACT ≥ 300 as well as the time passed over 300 seconds were significantly higher in patients under VKA versus DOAC, and higher in DOAC versus controls without significant differences however.

There was a trend toward more ACT < 300 after being >300 in the group of DOAC versus VKA patients, but this was not different between DOAC and controls. Finally, the number of ACT or patients with ACT ≥ 400 was significantly higher in VKA versus DOAC and higher in DOAC versus control patients although with borderline values.

#### 4.2.1 Multivariate analysis

Variables related with groups in univariate analysis (weight, duration of the procedure, gender and glomerular filtration) were included in multivariate analysis. Total heparin requirement was still significantly lower in VKA patients compared to DOAC (OR 1.09, 95%CI 1.03‐1.16, *P* = .005), but did not differ between controls and DOAC patients (OR 1.006, 95%C 0.98‐1.03, *P* = .6).

### 4.3 Time evolution of ACT and heparin dosings

**FIGURE 1:**
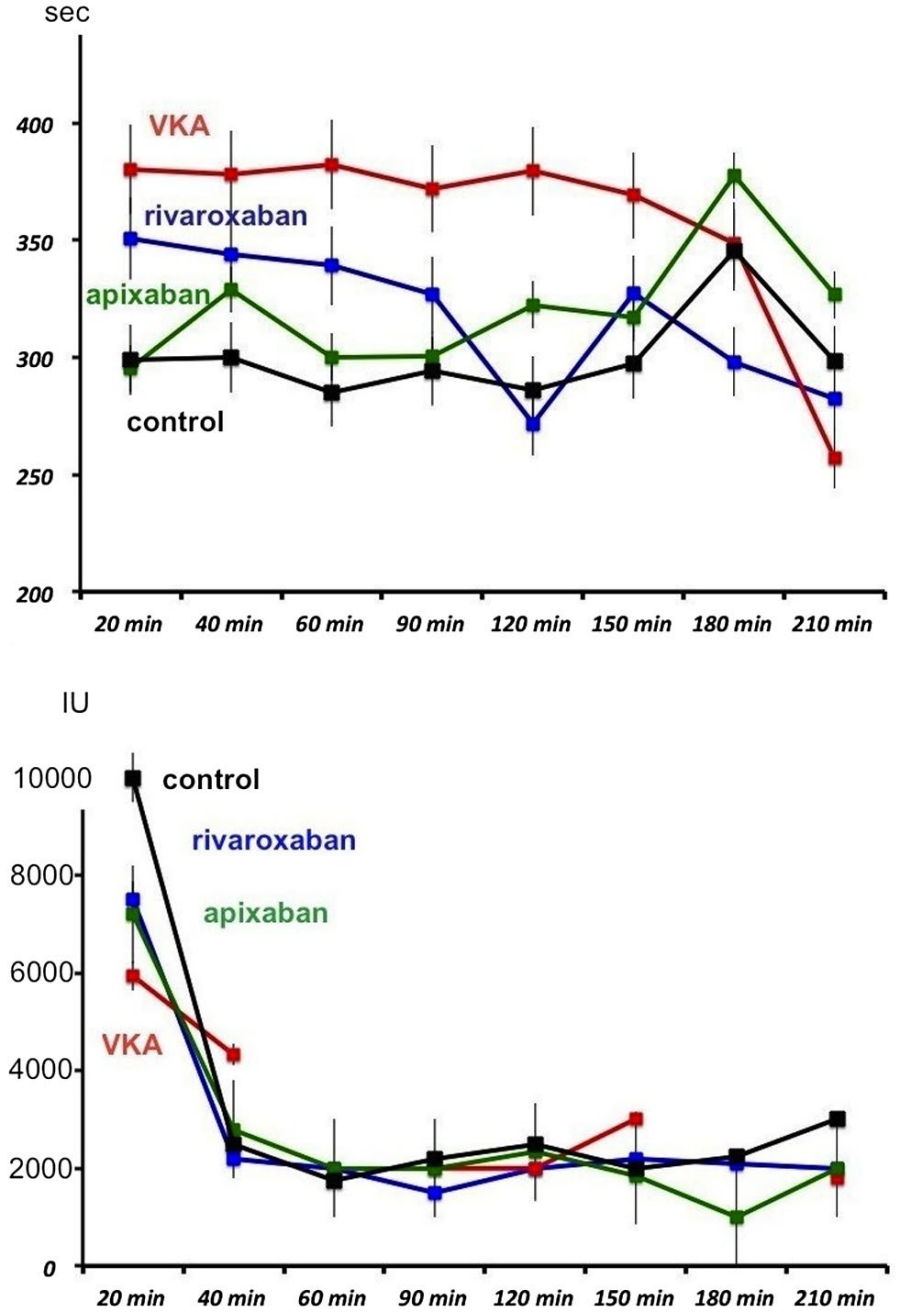
upper: evolution in activated clotting time (ACT) throughout the procedure in each group. ACT> 400 were coded as being measured for 450 seconds for graphical purposes. Lower: evolution in heparin requirements throughout the procedure in each group. Discontinuous line in vitamin K antagonists patients is explained by the lack of heparin need in any patient at some times

Evolutions of ACT and heparin dosings in the different groups during the procedure are shown in Figure 1. For ACT, the changes over time were not significantly different according to VKA, DOAC and control groups (*P* = ns). For evolution of heparin dosings over time, the number of values was not sufficient to perform statistical comparisons.

There was no vascular or thrombo‐embolic complication in any patient. Two control patients suffered from pericardial effusion (one needing percutaneous drainage) versus none in the other groups (*P* = .1). There was no other acute bleeding complication in any patient.

## 5 DISCUSSION

In this retrospective study on sixty consecutive patients undergoing percutaneous catheter ablation, our findings are as follows:

- Heparin requirements during the procedure were significantly lower in patients under VKA compared to DOAC, but were not significantly lower in DOAC patients compared to controls after correction of confounding variables.
- ACT values were significantly higher in patients under VKA compared to DOAC and similar in DOAC patients compared to controls. Furthermore, anticoagulation control (evaluated by the number/proportion of ACT ≥ 300 as well as the time passed over 300 seconds) was significantly better in patients under VKA versus DOAC, but the differences were not significant between DOAC versus controls. Finally, the number of patients/ACT with excessive ACT values was significantly higher in VKA versus DOAC patients versus controls.
- Except for longer baseline aPTT in patients with rivaroxaban versus apixaban, there was no significant difference between both drugs for any coagulation time or heparin dosing throughout the procedure.

Thus, in patients undergoing catheter ablation, long‐term therapy with VKA allowed less heparin requirement during the procedure despite reaching higher ACT values and more efficient anticoagulation control (although with a higher risk for excessive values) compared to patients under long‐term uninterrupted DOAC therapy which was similar to that of control patients. This may mean either that anticoagulation provided by DOAC + heparin is of lesser degree than those provided by VKA + heparin and similar to no anticoagulant, or that ACTs are not fully adapted to coagulation monitoring in patients on DOAC with heparin.

In previous large series however, no difference in embolic or bleeding complications had been noted between patients undergoing ablation procedures while on VKA or on uninterrupted DOAC therapy.^10^, ^11^, ^12^, ^15^, ^16^ In meta‐analysis, there was no significant association between bleeding events and ACT levels in patients receiving DOAC, while significant associations were found for embolic events.^17^ These uncertainties raise the question of the evaluation of anticoagulation level by ACT according to the type of oral anticoagulant drug. The optimal anticoagulation regimen for patients undergoing catheter ablation under DOAC will need additional studies including larger populations.

ACT is considered to evaluate the function of both the common and intrinsic pathways of the coagulation cascade by measuring the whole blood clotting time using a coagulation activator. ACT is known to show a strong linear relationship with therapeutic heparin levels and anti‐Xa activity of heparin, and is used for tens of years for monitoring of coagulation state promoted by unfractionated heparin in patients undergoing coronary angioplasty, extracorporal circulation or more recently catheter ablations.^3^, ^4^, ^5^

DOAC used in this study were both oral direct FXa inhibitors.^18^ At first glance there is no reason for ACT to not be able to monitor DOAC effect while being a reliable marker of heparin activity, because inhibition of factor Xa is the main effect of these drugs. However ACT has been showed to be poorly correlated with DOAC therapy, with large overlap between normal range and therapeutic DOAC levels.^18^ Low ACT values were reported at baseline in patients under DOAC or VKA with considerable overlap.^19^ At supratherapeutic concentrations, ACTs are consistently influenced by DOAC while therapeutic concentrations showed differential effects.^20^

ACT is known to efficiently evaluate the anticoagulation state provided by VKA + heparin, which has additive effects.^4^ However, the value of ACT in patients under DOAC has never been established and remains questionable. ACT may be inaccurate or inadapted with the use of DOAC and may be considered as a nonreliable surrogate for the overall anticoagulation status, especially after heparin administration, in patients with uninterrupted DOAC. Uninterrupted DOAC results in a large and unpredictable range of concentrations and highly variable level of anticoagulation at baseline.^21^ Alternatively, it is speculated that some DOAC such as apixaban may directly inhibit the level of FXa.^16^ Finally, ACT values on DOAC vary according to the device used and to the drug itself.^7^, ^22^ It is however unknown what are the correlations in patients treated by heparin.

Previous studies have investigated the differences in ACT and heparin requirements beween patients undergoing ablation of atrial fibrillation on VKA or truly uninterrupted DOAC, but none included controls.

A large retrospective japanese study included 859 patients on VKA or DOAC.^12^ Of note, half of the VKA patients were not correctly anticoagulated. Even if results were globally similar to ours, some differences happened between the different DOAC, rivaroxaban appearing superior in terms of coagulation control and apixaban needing the highest heparin requirements.

In a multicenter study, a greater amount of heparin was required in patients on uninterrupted apixaban versus VKA together with lower ACT values.^15^ In the VENTURE‐AF trial, higher doses of heparin were needed with lower ACT levels among patients with uninterrupted rivaroxaban versus VKA,^10^ while in the AFAXA‐AFNET trial, ACT was lower with apixaban compared to VKA.^11^ ACTs were significantly lower and heparin requirements were higher in patients under uninterrupted apixaban than VKA in another work.^16^ One meta‐analysis also showed that use of VKA was associated with reduced heparin requirements and with lower time to achieve the target ACT compared to DOAC either interrupted or uninterrupted.^17^

No control group does seem to have been ever investigated before. Our study is therefore the first one to compare ACT and heparin requirements between DOAC and controls, demonstrating that heparin requirements are similar for achieving similar ACT in patients with DOAC.

We did not find any significant difference between rivaroxaban and apixaban for any coagulation time or heparin dosing throughout the procedure. Concentrations of rivaroxaban have been suspected to be better correlated to ACT than apixaban with or without heparin,^7^, ^22^, ^23^ and rivaroxaban achieved better coagulation control and apixaban needed higher heparin requirements in the retrospective japanese study.^12^ Other works are needed to confirm or infirm these differences.

Potential solutions for performing ablation procedures with a more reliable coagulation control would be to switch patients to VKA, to interrupt DOAC (but this is not advised by the guidelines), to have more adapted point‐of‐care testings, or to use ACT after ex‐vivo reversion of DOAC.^4^

Even if prothrombin time‐INR may indicate higher exposure to rivaroxaban,^24^, ^25^, ^26^ this can not be used as a reliable marker of anticoagulation levels, and prothrombin time and aPTT are not recommended for monitoring apixaban.^16^ Ecarin clotting time, ecarin chromogenic assay, dilute thrombin time, prothrombin time with specific reagents, dosage of prothrombin fragment 1 + 2, anti‐FXa activity, calibrated drug‐specific anti‐Xa levels, or anti FII activity are either not routine, time‐consuming and/or expensive, but would be very interesting if point‐of‐care testings are available in the future.^16^, ^27^, ^28^

## 6 LIMITATIONS

We did not measure baseline ACT. This would have maybe demonstrated baseline differences between groups,^12^, ^19^ possibly explaining some of the differences noted thereafter during the procedure. However baseline ACT has been shown to be in the normal range with slight differences between some DOAC and VKA.^19^

Because more rarely prescribed in our area, dabigatran was not used in this study. Thus no speculation can be made for this drug.

## CONFLICT OF INTEREST

Authors declare no conflict of interest for this article.
